# Editorial: Leprosy reactions: New knowledge on pathophysiology, diagnosis, treatment and prevention

**DOI:** 10.3389/fmed.2022.1072274

**Published:** 2022-11-01

**Authors:** Roberta Olmo Pinheiro, Patricia Sammarco Rosa, John S. Spencer, Cleverson Teixeira Soares

**Affiliations:** ^1^Leprosy Laboratory, Oswaldo Cruz Institute, Fundação Oswaldo Cruz, Rio de Janeiro, Brazil; ^2^Research and Trainning Department, Lauro Souza Lima Institute, São Paulo, Brazil; ^3^Department of Microbiology, Immunology and Pathology, Colorado State University, Fort Collins, CO, United States; ^4^Pathology Department, Lauro Souza Lima Institute, São Paulo, Brazil

**Keywords:** leprosy reactions, diagnosis, treatment, neural damage, immunomodulation

Leprosy reactions may occur during the clinical course of the disease and are associated with an improvement in neural damage that contributes to the deformities and incapacities due leprosy (1–4). Early diagnosis of leprosy and the identification of prognostic factors related to the outcome of leprosy reactions are pivotal for reducing morbidity related to the disease (5–7). The mechanisms related to reactional outcome remain unknown. This is the central idea behind this Research Topic. Here, we selected outstanding papers that evaluated aspects related to early diagnosis of Hansen's disease, predictive markers of reactions and aspects related to diagnosis and treatment.

There is no gold standard test for leprosy diagnosis and the difficulty in distinguishing subclinical or asymptomatic infected individuals from those exhibiting active disease makes leprosy diagnosis essentially based on well-defined clinical signs and symptoms. Clinical examination is important to find out if the patients have any signs of skin or nerve damage. Slit skin smear (SSS) and histopathology are simple and important but require well-trained professionals and may not be available in resource constrained settings. The molecular diagnosis appeared as an adjunct strategy and the literature shows the efficacy of PCR of *M. leprae* DNA in difficult-to-diagnose cases. In this context, Lima et al.(a) measured the accuracy and performance among SSS and PCR of dermal scrapings stored on filter paper and anti-PGL-I serology for leprosy diagnosis showing that PCR combined with serological tests allows for a more sensitive and accurate diagnosis when compared to SSS alone. Since there are not many satisfactory immunoassay methods for leprosy diagnosis, Lima et al.(b) evaluated the use of serology against the mammalian cell-entry 1A (Mce1A) protein that is present in the cell wall of *M. leprae* and is associated with the entry of the bacillus into nasal epithelial cells and skin cells. They demonstrated that the Mce1A antibody profile can be an excellent diagnostic and therapeutic follow-up method to be used in Hansen's disease. Although serological tests using PGL-I have been used with limitations as a positive test cannot be used as a stand-alone diagnostic test, Antunes et al. demonstrated that anti-PGL-I serology at diagnosis is the most important prognostic factor for leprosy reactions after starting MDT. In this scenario, these data offer knowledge that can be applied in the development of new diagnostic strategies.

Clinical diagnosis of the neural forms of the disease is a challenge. Pure neural leprosy (PNL) is a clinical form in which dermatological signs are absent, but it is less well-understood than the dermatological forms of the disease. Pitta et al. evaluated the occurrence of reactions in PNL patients from a leprosy reference center as well as the occurrence of neuropathic pain. They demonstrated that PNL patients have more neuritis than those with classical leprosy skin reactions and that there is no association between acute neuritis and neuropathic pain. In another perspective, Feitosa et al. described that the pain that occurs in both leprosy reactional patients and patients with fibromyalgia may be a challenge in primary health care and although the leprosy reactional state is not a risk factor for fibromyalgia it can act as a confounder. The correct diagnosis of neuritis and leprosy neuropathy is essential to reduce disability and it certainly has an impact on public health.

The infection of peripheral nerves and neural damage, especially in response to reactional episodes, are hallmarks of leprosy. However, although there is no consensus about the involvement of the bacilli in neural damage, Junqueira de Souza et al. demonstrated that viable and dead bacilli differentially modulate the biology of Schwann cells, which can have implications for the ongoing neuropathy seen in leprosy patients. The understanding of the mechanisms associated with the neural damage will contribute to the development of more effective strategies of control for leprosy neuropathy and its complications. In this context, Pena et al. described the armadillo as a model for *M. leprae*-induced peripheral nerve injury that can provide insights toward the understanding of nerve function impairment progression.

Type 2 reaction or Erythema Nodosum Leprosum (ENL) is an acute and systemic inflammatory episode that may affect patients with the multibacillary form of leprosy. The pathogenesis of ENL is not fully understood and Rosa et al. performed an RNAseq to evaluate the overall gene expression in samples from ENL patients. They demonstrated that type 1 interferon is associated with ENL pathogenesis and that thalidomide, a drug used for ENL management in Brazil, can modulate the expression of several genes of the type 1 IFN pathway, suggesting that this pathway may be targeted for the design of specific, safer, and effective drugs against ENL. In order to elucidate the adaptive immune pathways associated with ENL, Gomes de Castro et al. characterized phenotypically and functionally CD4+ an CD8+ T cells *ex vivo*, comparing cells from non-reactional multibacillary patients with patients with type 1 or type 2 reactions. They observed a decrease in CD4+TGF-β+ Treg and CD8+TGF-β+ Treg in leprosy multibacillary patients during both reactional episodes, suggesting that the onset of reactional episodes involves the downregulation of Treg cells.

Thalidomide is teratogenic and some patients present a chronic and severe ENL which represents an extra challenge for the clinicians. Mendes et al. described their experience with four patients in which anti-TNF therapy was used with successful results, suggesting that anti-TNF may be used as an alternative in patients with chronic and severe ENL who do not respond to traditional treatment.

Reactional episodes are complex, but the understanding of the mechanisms associated with the onset and management of leprosy reactions is pivotal to reduce the morbidity associated with the disease (8, 9). Here, we discuss different aspects regarding reactional episodes, since diagnosis, immunomodulation, therapeutics, and new models to study nerve function impairment are linked to understanding and treating these debilitating episodes. All 10 papers published highlighted certain aspects of leprosy reactions but also point to the potential gaps in our understanding of the mechanisms associated with the establishment of leprosy reactional episodes.

## Author contributions

All authors listed have made a substantial, direct, and intellectual contribution to the work and approved it for publication.

## Funding

We thank FAPERJ [E-26/201.176/2021 (260734)], Fiocruz (INOVA Geração de conhecimento - 52301630878), and CNPq (312802/2020-0) funding institutions for all their financial support.

## Conflict of interest

The authors declare that the research was conducted in the absence of any commercial or financial relationships that could be construed as a potential conflict of interest.

## Publisher's note

All claims expressed in this article are solely those of the authors and do not necessarily represent those of their affiliated organizations, or those of the publisher, the editors and the reviewers. Any product that may be evaluated in this article, or claim that may be made by its manufacturer, is not guaranteed or endorsed by the publisher.
